# Operational challenges that may affect implementation of evidence-based mobile market interventions

**DOI:** 10.1186/s12889-022-13207-8

**Published:** 2022-04-16

**Authors:** Christina M. Kasprzak, Anne E. Lally, Julia J. Schoonover, Deanna Gallicchio, Lindsey Haynes-Maslow, Leah N. Vermont, Alice S. Ammerman, Samina Raja, Laurene Tumiel-Berhalter, Jill N. Tirabassi, Lucia A. Leone

**Affiliations:** 1 Department of Community Health and Health Behavior, School of Public Health and Health Professions, University at Buffalo, Buffalo, NY USA; 2 Department of Anthropology, The College of Arts and Sciences, University at Buffalo, Buffalo, USA; 3 Department of Sociology, The College of Arts and Sciences, University at Buffalo, Buffalo, USA; 4 Department of Exercise and Nutrition Sciences, School of Public Health and Health Professionals, University at Buffalo, Buffalo, USA; 5 Department of Agricultural and Human Sciences, North Carolina State University, Raleigh, USA; 6 Department of Nutrition, Gillings School of Global Public Health, University of North Carolina at Chapel Hill, Chapel Hill, USA; 7 Department of Urban and Regional Planning, School of Architecture and Planning, University at Buffalo, Buffalo, USA; 8 Department of Family Medicine, Jacobs School of Medicine and Biomedical Sciences, University at Buffalo, Buffalo, USA

**Keywords:** Food access, Mobile market, Programs, Low-income, Practices, Qualitative, Diet, Implementation, Barriers, Consolidated framework for implementation research (CFIR) stakeholders

## Abstract

**Introduction:**

Mobile produce markets are becoming an increasingly prevalent, accepted, and effective strategy for improving fruit and vegetable (F&V) access and consumption across underserved and lower-income communities. However, there is limited published research on mobile market operations. The goal of this research is to identify the challenges mobile markets face and ways to potentially mitigate those challenges. We will also discuss implications of our findings for future implementation of evidence-based food access interventions.

**Methods:**

We conducted 21 semi-structured key informant (KI) interviews to assess common practices of mobile market organizations that had been operating for 2 + years. We asked KIs about their organizational structure, operations, procurement and logistics, evaluation efforts, marketing and community engagement, success and challenges. A primary qualitative analysis involved deductive coding using qualitative software. A secondary qualitative analysis identified subthemes related to common challenges and remedial practices. A deductive coding process was applied to match identified challenges to the appropriate Consolidated Framework for Implementation Research (CFIR).

**Results:**

The leading challenges cited by KIs correspond to the CFIR domains of inner setting (e.g., funding and resources), outer setting (e.g., navigating regulations), and process (e.g., engaging community partnership). Practices that may mitigate challenges include maximizing ancillary services, adopting innovative volunteer and staffing structures, and formalizing agreements with community partners.

**Conclusion:**

Common and persistent challenges ought to be addressed to ensure and enhance the positive public health impacts of mobile produce markets. Contextual factors, particularly organizational factors, that impact implementation should also be considered when implementing an evidence-based intervention at a mobile market. Further research is needed to determine which innovative solutions are the most effective in mitigating challenges, improving implementation, and enhancing sustainability of mobile markets.

## Background

Mobile produce markets, or mobile markets, travel to predominantly low food access and lower-income communities to sell fruits, vegetables and other healthy foods [1, 2]. Research indicates that mobile markets are a promising solution for increasing consumption of fresh fruits and vegetables (F&V) [1, 3–7]. Furthermore, among food access programs (e.g., community supported agriculture, healthy corner store programs), mobile markets are a favored program among lower-income communities if they are conveniently located and sell affordably priced produce [8–10]. However, building awareness about mobile markets, their mission, and establishing trust among residents can be limiting factors to being accepted [10].

Compared to typical “brick and mortar” retail stores, mobile markets have the advantage of being flexible in where they locate and can adapt to changing food environments [11]. In addition, mobile markets are often managed by mission-driven, nonprofit organizations or residents and may have a deeper understanding of the communities they visit [2, 11–13]. However, many mobile markets face challenges with sustainability within the organization and the communities they serve [2, 14]. There is little research on mobile market practices which limits our understanding of the conditions that need to be met in order to be effective (e.g., increased access, F&V consumption). To fill this gap, we started with the objective to identify common practices for mobile markets with an eventual goal of establishing standards of practice to tailor to the needs of different organizations and communities [2].

There is scant research on the processes of designing, operating, and sustaining mobile markets from the perspective of the organization. The available research indicates that issues surrounding financial sustainability, lack of organizational capacity, and difficulty attaining community buy-in can undermine the mobile markets' reach, impact, and longevity [12, 13]. There is a need to understand the persistent challenges that may undermine an organization’s capacity to implement a mobile market so we can ensure programs are using optimal practices.

Understanding contextual factors that either help or hinder mobile market operations is crucial in facilitating implementation and ensuring interventions have optimal public health impact. A review of over 500 studies evaluating prevention and health promotion programs found that the influence of better implementation (e.g., fidelity, dosage, reach) on health outcomes has resulted in mean effects sizes that are two to three times higher in treatment groups compared to controls [15]. Therefore, implementation influences outcomes; in the case of mobile markets, this can translate to a reduced impact on dietary changes (i.e., F&V intake). Specifically, implementation is mostly impacted by variables related to communities, implementers, intervention, organizational characteristics, and available support systems (i.e., training and technical assistance) [15]. Identifying common challenges experienced by mobile market organizations is the first step in understanding the contextual factors that may impede adoption and implementation of mobile market programs by community-based organizations.

The purpose of this research is to raise awareness of the challenges that mobile markets face and encourage researchers, policymakers, funders, and stakeholders to offer their support in the areas of greatest need. Understanding innovative strategies can also provide a precedent for organizations to adopt, allowing them to circumvent potential challenges. The current study furthers existing research by focusing on mobile markets operating at least two years and identifying practices that may ameliorate challenges.

Through in-depth interviews with mobile market organizations, we seek to answer the following: 1) What are the challenges established mobile markets commonly face? 2) What are the implications of these challenges for future implementation of evidence-based interventions? and 3) What are the practices that potentially mitigate operational challenges?

## Methods

### Recruitment and enrollment

**Fig. 1:**
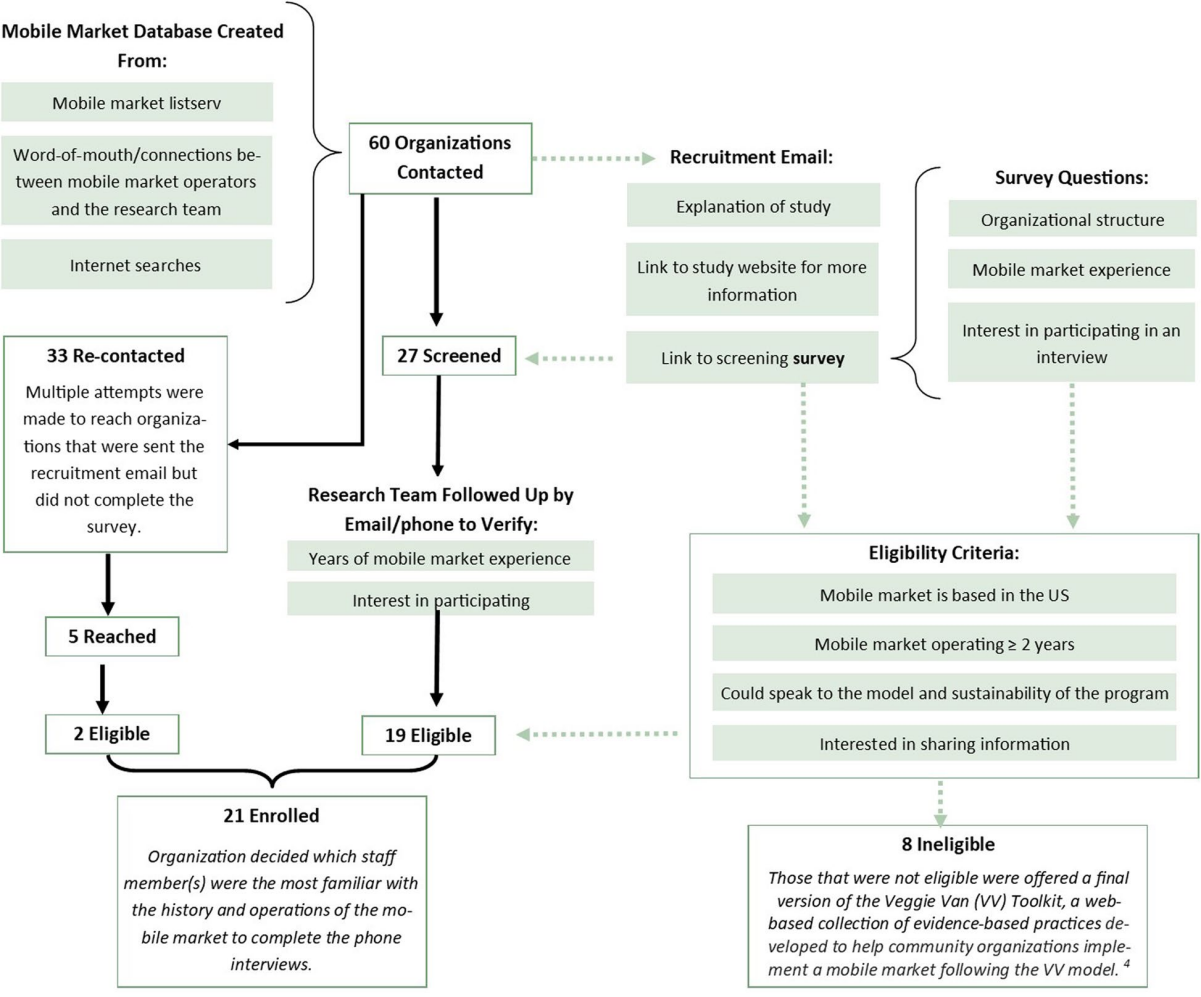
Key Informant Recruitment and Enrollment Process

Figure 1 depicts the key informant recruitment and enrollment process.

### Key informant interviews process

Semi-structured interviews were conducted over the phone between May and November 2018. The interview guide was informed by the research team’s collective experience operating and evaluating mobile markets. The initial interview was approximately 90 min and key informants (KI) were asked questions regarding market models, staffing, nutrition education and ancillary services, business and financial models, logistics and operations, community engagement and marketing strategies, procurement and pricing, and program impact and evaluation. Organizations recognized as possessing a certain strength or uniqueness were asked to complete a follow-up interview, for a total of four calls maximum. Although the focus of the interview guide was to assess common practices among mobile market organizations, persistent challenges emerged as a dominant theme throughout data collection and analysis, leading us to generate a separate research question from the original research aim.

In addition, several practices that help ameliorate challenges among mobile market organizations emerged as a theme in the original formative work generating an additional research question that compliments the findings on challenges. The findings from the original research question, specifically the common practices and important resources among mobile markets organizations, are reported elsewhere [2]. KIs were compensated $50 for each interview they completed and offered access to the Veggie Van (VV) Toolkit, a web-based evidence-based program intended for mobile markets. This study was approved by the University at Buffalo Institutional Review Board and all methods were performed in accordance with the relevant guidelines and regulations.

### Data analysis

The primary data analysis is reported elsewhere. Briefly, the original research aim was to identify common practices through a deductive analysis utilizing a pre-established codebook. Code reports and memos from that research underwent a secondary analysis in Microsoft Word and Excel to identify subthemes related to common challenges and remedial practices. Lastly, a deductive coding process was applied to match identified challenges to the appropriate Consolidated Framework for Implementation Research (CFIR) construct (e.g., staffing issues; domain: inner setting) to assess organizations’ pre-implementation capacity.

### Conceptual framework (CFIR)

Conceptual framework (CFIR) was utilized for the secondary analysis of qualitative data. CFIR was developed in 2009 by Damschroder et al. in response to a call for a greater use of theory to guide implementation research [16, 17]. CFIR was a chosen framework due to its flexibility to be used across the spectrum (pre-, during, post-implementation) of implementation, the inclusion of the most salient implementation factors, its strong theoretical foundation, and the ease in which it can be tailored to different interventions and settings [16–18]. CFIR is comprised of 39 constructs within five major domains that interact to influence implementation of programs and interventions and their eventual effectiveness [16].

## Results

### Organizational demographics

Twenty-one mobile markets were represented by 25 KIs in interviews and no participants withdrew from the study once enrolled. Table 1 includes characteristics of the participating mobile market organizations. The KIs were all mobile market staff (e.g., director, market manager) and represented organizations from 16 states and 19 cities in the U.S. The majority of mobile markets serve predominantly or exclusively urban areas and are managed by a non-profit organization.

**Table 1:** Mobile Market Organization Characteristics

**Region of United States**	**Number of Mobile Market Organizations**	**Organizational Structure**	**Percentage of Mobile Markets (n)**
Northeast	10	Non-Profit (Other)	48% (10)
South	6	Non-Profit (Hunger relief/Food Bank)	14% (3)
West	3	Non-Profit (Hospital Network)	10% (2)
Midwest	2	Stand-alone Mobile Market Non-profit	10% (2)
**Years Operating**	**Percentage of Mobile Markets (n)**	Non-Profit (Foundation)	5% (1)
3 years	19% (4)	Non-Profit (Public Health Entity)	5% (1)
4 years	29% (6)	University/College	5% (1)
5 years	19% (4)	City/Municipality	5% (1)
6 years	5% (1)	**Region Served**	**Percentage of Mobile Markets (n)**
7 years	19% (4)	Urban	67% (14)
8 years	5% (1)	Mixed (urban/rural/suburban)	24% (5)
9 years	5% (1)	Rural	9% (2)

**Fig. 2:**
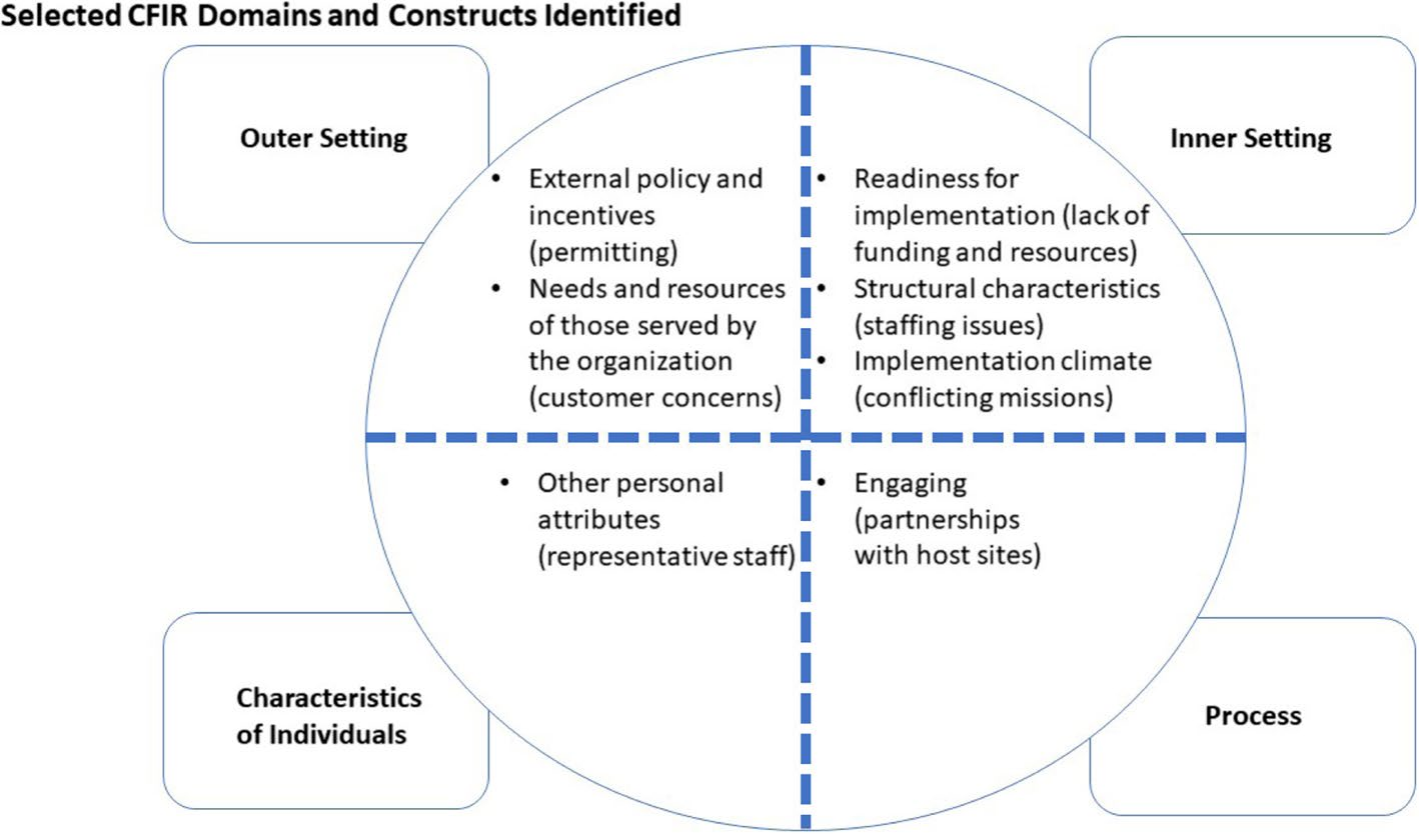
Selected CFIR Domains and Constructs Identified

Our findings on common challenges are reported below, organized by the CFIR domain, construct, and sub-construct (if applicable). Figure 2 depicts the CFIR domains and constructs that were identified.

### Domain/construct: outer setting/needs and resources of those served by the organization

challenges that are associated with external influences on mobile market operations fall within the domain of “outer setting.” within this domain, the challenges cited correspond to the construct “needs and resources of those served by the organization.” Organizations commonly collect non-sales data (e.g., demographics, customer feedback) and many of the organizations have gone through some form of evaluation. The scope and rigor of the evaluations are highly variable and can be conducted internally by the organization (e.g., program evaluator) or in partnership with an outside organization such as a local university. KIs expressed concern surrounding data collection in regard to burdening their customers. Specifically, organizations do not want to jeopardize the integrity and privacy of their customers or risk damaging the relationship and trust they have established.

*“It's virtually impossible to do that (data collection) …our funding in the past for these things has been through health organizations and we found that that kind of tracking, it's not accurate, first of all, and it's just really, really hard to actually collect and … it alienates customers." Oregon Mobile Market Key Informant*

### Domain/construct: outer setting/external policy and incentives

Within the domain of “outer setting,” the construct “external policy and incentives” includes challenges related to federal and local regulations. Nearly all organizations participate in at least one type of incentive program, usually a Supplemental Nutrition Assistance Program (SNAP) matching program; however, there are barriers to enrolling in the Women, Infants, and Children (WIC) and Seniors Farmers Market Nutrition Program (FMNP). Organizations that are unable to achieve the necessary classification (e.g., farm stand) or procure the minimum percentage of local produce are ineligible to participate in these programs. In addition, many organizations face regional barriers regarding food handling requirements that prevent or greatly hinder the ability to conduct food demonstrations at the market, which are a preferred form of nutrition education.

### Domain/construct: inner setting/structural characteristics

The majority of the challenges cited by KIs fall within the CFIR domain of inner setting, which includes implementation factors related to the structure of an organization which corresponds to the construct “structural characteristics.” KIs expressed that operating a mobile market is an “expensive business model” due to not being able to break-even and cover operating costs solely through sales revenue. The leading expenses that were mentioned by KIs were the cost of produce, staffing, and the vehicle and related expenses (e.g., repairs, fuel). The percentage of operating costs covered by sales ranged from 10–75%, requiring organizations to seek additional funding sources. Supplemental funding comes from a combination of sources including national and regional grants (*n* = 19), support from parent (e.g., food bank, city/municipality) and/or partner (e.g., health department or network) organization (*n* = 8), foundation funding (*n* = 7), corporate sponsorship (*n* = 6), fee-for-service events (*n* = 2), and philanthropy and donations (*n* = 3). The pressures of acquiring and maintaining funding to support mobile market operations are persistent challenges among organizations. Most KIs expressed it is difficult to be self-sufficient because selling produce at a low cost reduces profitability, making them dependent on supplemental funds.

*“I think another challenge is having long term funding to staff the program, whereas I said we're working towards increasing our program generated revenue. But it's an expensive business model and we're not close to covering our costs. I don't think that we will get there at [that] scale anytime soon and so having long term funding to support the staffing to run this program, is huge.” Massachusetts Mobile Market Key Informant*

KIs commonly mentioned staffing challenges; market staff levels are highly variable across organizations, with KI’s reporting 0–8 full-time staff, 1–6 part-time staff, and 1–30 volunteers within the parent organization at-large and 1–3 paid and 1–5 volunteers for the direct mobile market staff. Staff are often shared with other programs run by the parent organization, work in administrative roles related to the market, or complete a multitude of direct market tasks (e.g., running the market, cashing out customers, driving the vehicle). In addition, the seasonal nature of most positions and the lack of financial means to pay staff as much as organizations would like increases staff turnover. Although volunteers are a valued asset among organizations, the very nature of these unpaid, often temporary, positions can make them more of a liability than a benefit. Therefore, some organizations limit their numbers to a core group of reliable volunteers to avoid misallocating training resources.

### Domain/construct: inner setting/implementation climate

Within the domain of “inner setting” challenges that relate to an organization’s capacity and receptivity for change and flexibility correspond to the construct “implementation climate.” Within this construct, challenges related to upholding an organization’s values fall within the sub-construct of “compatibility.”

#### Sub-construct: compatibility

All organizations prioritize sourcing locally, but other factors need to be considered when making sourcing decisions, which presents challenges for reconciling missions (i.e., support local farmers vs. sell low-cost produce). For example, the climate or geography of a region may result in certain produce to be prohibitively expensive to purchase because the organization would either be forced to price out of their customers’ means or take a complete loss over the sale. Organizations tend to prioritize affordability and therefore may look to other produce sources (e.g., regional, neighboring state, import) if it ensures that they can meet customers’ needs while remaining financially viable. In addition, some organizations are open to sourcing imported produce to provide culturally relevant produce (e.g., plantains, yucca root) that increases customer acceptability. However, a few organizations have a more inflexible overarching mission (e.g., support local farmers) that governs sourcing decisions even when wholesale prices increase, or customers request non-local produce. These organizations may receive pushback from customers regarding the lack of imported produce, but see this as an opportunity to educate the community about the importance of sourcing locally. The push-and-pull between conflicting factors may lead organizations to compromise their priorities for supporting local agriculture, honoring customers’ cultures, or achieving financial sustainability.

*“During the growing season we source from local farmers as much as we can but it’s challenging because the cost of the food is higher with local farmers…. [the percentage from local farmers has been] higher in past years but we kept losing money. So, we had to make a change unfortunately, it breaks my heart to do it.” Minnesota Mobile Market Key Informant*

### Domain/construct: inner setting/readiness for implementation

The construct “readiness for implementation” includes challenges that may undermine an organization’s commitment and capacity to implement an intervention. The sub-constructs “available resources” and “access to knowledge and information” include challenges associated with the level of resources available and the ease of access to information and knowledge related to mobile market operations and implementation, respectively.

#### Sub-construct: available resources

Most organizations utilize 1–2 trucks, vans, or busses to transport produce to market sites. Although most set up the market on the vehicle's perimeter or within the host site (i.e., community partner hosting the market), organizations retrofit the vehicle(s) to suit their specific needs (e.g., storage, refrigeration). The expenses associated with the initial vehicle purchase, upgrades, and maintaining the vehicle(s) are a costly and ongoing challenge. Finding qualified experts to service and retrofit the vehicle(s) can be difficult, particularly with de-commissioned vehicles that require a particular expertise (e.g., transit or school bus). The inconvenience from a disabled vehicle deeply impacts the market patrons and the viability of the market due to lost sales.

*“…there’s always a crisis… whether it’s the bus breaking down or the walk-in cooler going out…” Minnesota Mobile Market Key Informant*

All organizations have access to dry and cold storage at their operations’ hub or nearby storage; however, some KIs expressed their organization’s storage is not ideal due to inadequate or shared space. Most organizations invest in some type of refrigeration (e.g., coolers, refrigerators, Cool-bot system) for the vehicle and/or their hub; many mention that a refrigerated truck would be optimal, yet out of reach for most.

Organizations are largely satisfied with their current sales tracking methods, with most using Point-of Sale (POS) software (e.g., Square ®) compared to handwritten sales ledgers. However, some feel that their current sales tracking system is outdated, but costs limit their ability to upgrade. Chosen POS software may not be ideal for capturing and viewing data, particularly when processing incentive program transactions, and organizations would like a more functional and streamlined platform.

Depending on the market setup and weatherization, extreme temperatures can impact customer comfort, produce quality, and functionality of POS technology. For example, a market enclosed in a vehicle where customers enter to shop may still be too cold for customers. Alternatively, a market that operates outside of the vehicle may experience wilting produce and malfunctions with POS technology due to extreme heat.

Most of the organizations work with partner organizations (e.g., local Extension office, local college nutrition students/interns) to offer regular nutrition education. Organizations offer their own informal nutrition education such as distributing recipes, but due to staffing and time constraints, more comprehensive education (e.g., mini lessons, cooking demonstrations) are typically left to the partner organizations. Many organizations would like to offer more nutrition education activities but are unable to do so for the aforementioned policy and staffing barriers along with a lack of space.

#### Sub-construct: access to knowledge and information

The majority of market sites are chosen based on partnerships with community sites that serve a similar target market as the mobile market organization (e.g., low-income housing, libraries, community centers, etc.). While the breadth of sites is largely the same between organizations, there is variability in the types of sites that are busiest. Therefore, predicting which potential market sites will be viable is an ongoing challenge as is optimizing the mobile market schedule.

Prices are often set informally based on trial-and-error or comparing prices to local retailers. This approach typically leads to a retail price that is a 10–20% markup over wholesale cost. However, it can be challenging to set price points on perishable items that regularly fluctuate in cost and availability while ensuring affordability and financial sustainability. KIs expressed interest in establishing a better and easier pricing system. Organizations would also like to improve their inventory management to avoid produce shortages and minimize waste.

Among organizations that are able to provide their own form of nutrition education, KIs underscore the importance of culturally relevant education. However, some lack the means (e.g., translation services) to tailor their education to different languages and cultures.

The most common marketing strategies to build awareness and interest in the market include print (e.g., flyers, signs), social media (e.g., Facebook), broadcast (e.g., TV, radio), and digital media (e.g., email, text). Word-of-mouth, networking, and canvassing are commonly cited as more successful strategies. However, few organizations have a formal marketing plan or track their marketing efforts, and therefore can only speak to the effectiveness of their strategies anecdotally.

### Domain/construct: characteristics of individuals/other personal attributes

The domain “characteristics of individuals” reflects the qualities of the individuals within an organization; the construct “personal attributes” includes individuals’ personality traits. KIs expressed that staff are expected to “wear a lot of hats,” and this presents a challenge in finding diverse and qualified employees that are suited for working at a mobile market. Subsequently, this can lead to staff burnout and high turnover rates. Organizations also struggle with hiring staff that are representative of the communities that the mobile market serves.

*“I think running a program like this is somewhat unusual just in the diversity of functions that everybody is doing. So, like, even when we hire market staff…we need people who are comfortable with physical labor, and driving a truck, and also incredibly charming, and good at customer service which aren't always the same people.” Rhode Island Mobile Market Key Informant*

### Domain/construct: process/engaging (external change agents, innovation participants)

The domain “process” includes constructs related to the process of implementation; the construct “engaging” refers to attracting and involving the appropriate individuals throughout implementation. The sub-construct “stakeholders/external change agents” includes engagements with influential individuals outside of the implementing organization. The sub-construct “innovation participants” includes engagements with those served by the organization (e.g., mobile market customers).

#### Sub-construct: engaging stakeholders/external change agents

Most organizations have an informal agreement with host sites, while some draft a memorandum of understanding (MOU) or similar contract with community partners. Organizations adjust their expectations of partners’ involvement in the mobile market based on each site’s capacity; however, KIs expressed that a lack of clarity on the roles of partners can lead to frustration. Some organizations would like to outline more robust expectations and encourage partners to take a more active role in supporting the market (e.g., promotion, provide space).

*“I felt like there are – the role of partners wasn’t clear. And so, I think that causes some frustration with the collaborative. Just not clear expectations of roles.” New Mexico Mobile Market Key Informant*

Organizations procure produce from a combination of sources, but predominantly source directly from farms. However, securing an agreement with a farm can be a challenge due to difficulties in identifying a farmer that is willing to work with a mobile market, as well as effectively negotiating for high quality produce at a fair price.

#### Sub-construct: engaging innovation participants

Most organizations are actively engaged with the communities they serve through attending community events, meetings, speaking engagements, and engaging with policymakers—but few have a community advisory board. Most are interested in forming or reviving a community advisory board specifically for the mobile market but have other competing priorities.

Most organizations feel they are adequately reaching their target market, but this belief is mainly based on sales data that indicates what percentage of customers receive SNAP benefits. Organizations are less confident in assessing their reach with subsets of the lower-income population, such as individuals that do not participate in assistance programs but still experience food insecurity. KIs expressed it can be a lengthy and delicate process to earn trust when expanding the mobile market to new communities. There may be confusion among community members surrounding whether the mobile market is a charity, a service, or both. KIs underscored the importance of persistence and patience for community members to accept the concept of a mobile market and to cultivate trust. Earning recognition and buy-in from community partners and government entities can also be a challenge.

*“…literally it took six months before we had customers. Every single week for an hour [for] six months, but if you think about it, it's a rough neighborhood [and] they don't trust anybody, ‘who is this new person?’ …. So it's again you have to show up every single week and you have to say hello to every single person that walks by.” Michigan Mobile Market Key Informant*

#### Biggest challenges faced by mobile markets

KIs were asked which of the challenges that organizations face in operating a mobile market are the most formidable. The most frequently cited challenges were related to staffing, funding and financial sustainability, effectively engaging with community partners and residents, vehicle acquisition and maintenance, and weather conditions.

*“Definitely the biggest for us is staffing, keeping [staff] … One of the reasons why I think our program is successful is because of our customer experience. So, customers come to us and they have a really positive experience and part of that is having enough staff there to make it work and to keep it a positive place…And that’s always tough that’s the biggest part of our budget is staffing.” Pennsylvania Mobile Market Key Informant*

#### Practices that may address common challenges

Organizations employ numerous practices that may help to mitigate persistent challenges. Table 2 lists linkages between innovative practices and common challenges that were reported. Strategies aimed at soliciting investment, maximizing profits from other services, or adopting a cost-offset pricing model may reduce challenges related to available resources and sustainability. Restructuring staffing models to rely more heavily on volunteers, community champions, or implementing a “train the trainer” model (i.e., train community members to provide nutrition education for the mobile market) may reduce challenges related to staffing and capacity. Forming strategic partnerships and extending the market season may facilitate engagement with communities and enhance program reach. Conducting formative work to understand community needs and establishing more formalized arrangements and expectations of host sites may strengthen community engagement and enhance the viability of sites.

**Table 2:** Practices that May Mitigate Common Challenges

**Challenges Addressed**	**Practices**	**Example**	**Illustrative Quote**
Expensive business model; lack of financial sustainability	Solicit investment in the mobile market to fund operations	Consistent philanthropic donations and gifts (e.g., donated vehicles); business/corporate sponsorship	What we've done successfully for the last number of years is we've gotten all of our vehicles paid for by private philanthropy." *New York Mobile Market Key Informant*
Staff issues	Promote staff solidarity, communication, and empowerment to enhance employee retention and satisfaction	Offer a variety of professional development trainings for staff, holds weekly staff meetings, pays an hourly wage above the state minimum wage	" So, taking folks out to lunch at the end of the season, supporting them to go to trainings …that's not in the screening process that's more just in sort of having an eye toward retention and appreciation, which I think you see the payoff because you don't waste the energy in recruiting and training." *Massachusetts Mobile Market Key Informant*
Expensive business model; lack of financial sustainability; staff issues	Adopt innovative staffing models to facilitate market expansion without increased staff expenses	Shift staffing to a greater reliance on volunteers and a reduced reliance on paid staff	"So, we're piloting a few different things. One is the new staffing model…where we're going to be just having one paid staff per bus with staff as volunteers. So as soon as we can put that second bus into operation, we’ll have increased volume of sales without doubling our staffing expenses." *Minnesota Mobile Market Key Informant*
Expensive business model; lack of financial sustainability; staff issues	Participate in federal service programs that mobilize committed and mission-driven students for employment and share staff costs	Participate in an Americorps program (VISTA, National, State) and hosting students as employees	"It was a natural progression of our work to go from growing food and inviting people to the farm to realizing the barriers that our communities face and accessing food…which we address with our Mobile Farmers Market by having our AmeriCorps members who are trained, know how to cook the produce, how to grow the produce, answering questions about it…but the majority of our funding comes from AmeriCorps branch which is a federal grant program." *Maryland Mobile Market Key Informant*
Expensive business model; lack of financial sustainability	Maximize profits from other services	Commercial kitchen (small food business incubator), food hub, and community supported agriculture (CSA) shares; expand food production (e.g., high value crops) and/or aggregation and distribute to restaurants and institutions	"We have a shared commercial kitchen and food business incubator so that is basically a commercial kitchen facility that we've built to support new and existing small food businesses. So, they pay by the hour to rent our space and run whatever business it is they run." *Virginia Mobile Market Key Informant*
Expensive business model; lack of financial sustainability; community engagement issues	Scale up the profitability of events	When mobile market is invited to community and corporate events, charge a fee to corporate sites while extending a sliding scale cost or no charge to community sites	"For our popup events, our corporate groups pay the full cost of overhead for their events…as well as the overhead associated with a popup event in our target community. So that [is] $1,000 for them, $500 for their own event and $500 for sponsoring events in our neighborhoods. The folks in our neighborhood don’t have to pay for overhead; all they pay is the price of the food that they’re distributing." *Massachusetts Mobile Market Key Informant*
Staff issues; community engagement issues	Ensure that staff are representative of the demographic of the communities that the market visits	Prioritize hiring staff that reside in the communities served	"That lived experience is valuable to have as part of the team. Having also the lived experienced on being on SNAP or EBT or WIC knowing truly what it is like to try to get the quality food that you want in that area in your neighborhood; and also, being part of a solution and the joy that comes from that is some of the biggest feedback that I’ve gotten from staff that are from the community we are serving." *DC Mobile Market Key Informant*
Limited organizational capacity for nutrition education; reach/penetration of target market	Employ staff that are qualified to provide regular nutrition education	Employ staff, such as a nutritionist, that largely focus on cooking-related programming including nutrition education directly at the market	"[We] hired a community nutritionist and so he again helps make the connection that food is medicine and can talk to folks more specifically about their possible health challenges…he's been a farmer, he's worked for another hospital system the city and is bilingual, that's really important [for] our market to have at these Spanish speakers staffing…we pay him to be at the market." *New Mexico Mobile Market Key Informant*
Staff issues; community engagement issues	Recruit dedicated community members that serve as “champions” or “advocates” for the mobile market, acting as a liaison between the mobile market and the community	Champions may be connected to a host site (e.g., resident at a housing that hosts the market) or to the mobile market (e.g., regular customer) and typically assist with community engagement and marketing	"We're not getting as many young families and Latinos as we would like to see proportional to the makeup of the city, and so we've been doing targeted outreach at sites that have a higher concentration of those populations. We're piloting a resident champion model this year, so we hired someone from one of the large public housing facility sites…he’s a native Spanish speaker and he lives in that community. So, he's really focusing on targeted outreach." *Massachusetts Mobile Market Key Informant*
Staff issues; community engagement issues; procurement issues	Establish competitive and synergistic volunteer programs	Volunteers accepted into the work-share program receive credits toward produce at the market in exchange for hours worked (e.g., $10 of produce for every hour volunteered); residents from the communities served are given priority acceptance	"But we do have a lot of consistent volunteers, and folks that are signed up for our work shared programs, so community members can agree to serve on our mobile market or our farm and earn produce shares to use at the mobile market. " *Maryland Mobile Market Key Informant*
Community engagement issues; reach/penetration of target market	Establish partnerships with local service providers that actively engage with the same target market	Partner with the local WIC or Department of Social Services (DSS) offices; mobile market marketing materials are included in Farmers Market Nutrition Program (FMNP) benefit folders and SNAP recertification paperwork	"We did an outreach campaign with our WIC office….and then having them include our schedule with all of the pockets of the [Farmers Market Nutrition] coupons….that did lead to a significant increase in our WIC coupon utilization." *Massachusetts Mobile Market Key Informant*
Expensive business model; lack of financial sustainability	Expand reach beyond lower-income communities	Partner with supplemental sites that attract mixed or higher income customers	"Well, industry standards are...there's a minimum of 33% mark up on produce, and it goes up to [as] high as about 42%. So, I stay within that bracket [at corporate sites] …they're lower at the community sites. " *New York Mobile Market Key Informant*
Host site issues; community engagement issues	Establish explicit and formalized expectations of host sites	Host “lead team” meetings with host site contacts on a monthly basis that are focused on discussing market logistics	"So, we have a lead team that meets monthly throughout the year. And on the lead team several of the site contacts are the champions for the market and attend regularly. At times we've had subcommittees…an outreach subcommittee really was an informal couple of meetings where our site contacts would sit down with each other and talk about best practices." *New Mexico Mobile Market Key Informant*
Community engagement issues	Formation of advisory committees to guide decision making	An advisory committee comprised of community partners and residents meets quarterly to discuss strategic planning decisions	We have a broader advisory group. That’s made up of eight individuals, four which are community members and four of which are folks who work with organizations, that work directly with the communities that served. So, they do bigger picture work. " *New Mexico Mobile Market Key Informant*
Community engagement issues; site viability	Conduct formative work prior to market launch to inform decisions on site location and market planning	Conduct stakeholder interviews, listening sessions, and focus groups with community members	"So, we did a ton of community engagement prior to launching the program because we really wanted the mobile market to be informed and driven by the community, and so we did outreach and engagement with over 500 residents." *Minnesota Mobile Market Key Informant*
Site viability; host site issues	Formalize the process for identifying host sites	Implement an application process to ensure that site decisions are based on community need/demand and the applicant’s ability to partner	"So, they fill out the application and then we review the application in a similar manner maybe a funder would review a grant. And we make decisions based on need, and also the organization's ability to actually partner with us, meaning we bring the food and the education to the neighborhood but it's on them to build the community around it and get people to the stand." *New York Mobile Market Key Informant*
Community engagement issues; reach/target market penetration; site viability	Innovate and continually update market schedule and location	Rotate new and interested sites in a monthly rotational time slot as a means to pilot the site	"We have four different locations plugging into a monthly rotational spot. That's how [market site name] started out. They were a monthly site and then they were far away the busiest site every time, so the next year we gave them a weekly slot. " *New York Mobile Market Key Informant*
Site viability; host site issues	Explicit and formalized expectations of host sites	Set minimal criterion of the number of engagements per hour or produce sold per hour that a host site must guarantee	"We discuss the terms that they need to be doing for outreach. And we do put in the application that they either need to meet our sales minimums or our visitor minimums. And right now, we're at, I believe it's a $110 an hour or 40 people per hour, which sounds high but it's totally doable." *New York Mobile Market Key Informant*
Host site issues; community engagement issues; reach/target market penetration	Expect a greater degree of involvement in community outreach and market day logistics	Host sites may be asked to take on the brunt of the community outreach and marketing in the form of canvassing, phone or text reminders, social media posts, posting and distributing signs, and making PA announcements	"We rely on the sites primarily for outreach…we expect them, and some do it more passionately than others to just be using that word of mouth and that relationship to make sure that people know about the market." *Rhode Island Mobile Market Key Informant*
Host site issues; site viability	Implement a process for tracking and holding sites accountable	Provide regular feedback to host sites based on market performance (e.g., sales, reach, traffic); maintain a public facing scoreboard that all partners can view	"We’ve tried actually sending out like a public score board. It’s not ranked or anything, but it gives everybody a chance to see where they stand. And it gives us a little bit of weight when we’re having that conversation." *Massachusetts Mobile Market Key Informant*
Community engagement issues; expensive business model; lack of financial sustainability	Solicit investment in the mobile market	Encourage or expect host sites to invest in the mobile market by purchasing coupons/vouchers for their clients to spend at the market	"We'll often times go to some of our partners and say, 'All right, we're not charging you for this,' because we don't charge anyone for our attendance. But I'll say to some of them, give me a hundred bucks and I'll split it out into 50 $2 vouchers that we can then give to your clients, or your residents, or whatever the case may be. So, sometimes we'll ask our partners to actually invest in the program, but its money for the consumer use." *New York Mobile Market Key Informant*
Expensive business model; lack of financial sustainability	Expand inventory to include high profit items	Secure a sponsorship with large food companies that will donate non-produce stables (e.g., meat, eggs, milk) that the market can sell at a 100% profit	"We're always looking for creative food sourcing strategy. So right now, one of our sponsors is [Brand Name] and they donate the deli meat direct to us from their warehouse and then we turn on and sell it for 100% profit, but we sell it as significantly lower price than what people would pay on the store." *Minnesota Mobile Market Key Informant*
Procurement issues; expensive business model; lack of financial sustainability	Connection to a farm within parent organization which serves as a partial source of produce	Engage in some form of farming either on their own property or throughout the community	"I’d say 80 percent of the produce is from our farm. And then like the other 20 percent, I buy directly from like farms that I know. [Farm name] is a member of the Farm Alliance of Baltimore City, which has I think 12 or 15 active member farms." *Maryland Mobile Market Key Informant*
Procurement issues; reconciling missions; expensive business model; lack of financial sustainability	Establish relationships that facilitate procurement of local produce and minimize price	Establish and cultivate relationships with a food hub, aggregator, or agricultural cooperative/network	"So we buy from about 10 different entities. So, most of those are small independent farm and then one of those is an aggregator…she follows the same kind of hundred-mile radius that we do, but through her we can buy a wider variety of products, we can buy at wholesale prices a little more easily and get a lot of products that the independent farms that we work with don't grow or carry…she sources from about 50 local farms." *Virginia Mobile Market Key Informant*
Expensive business model; lack of financial sustainability	Establish and leverage relationships with local organizations to facilitate vehicle updates and maintenance	Utilize a mechanic that specializes in camper vehicles due to their similarities to mobile markets; partner with local college students in architecture or design programs to retrofit the mobile market as part of a class project	"We purchased a box truck…and sent it up to a nearby University, James Madison to the industrial design school where some seniors in that school took on retrofitting our truck as a as their kind of senior project, their senior kind of workshop project." *Virginia Mobile Market Key Informant*
Expensive business model; lack of financial sustainability	Adopt a form of differential pricing	Establishing prices based on the type of community site (e.g., lower-income versus mixed/higher income community) or the customer (e.g., SNAP-eligible versus SNAP ineligible)	"We set our retail price as close to double wholesale as possible and as reasonable…and so then full priced customers will pay retail and then anyone who received the [SNAP] match will pay essentially the wholesale cost…so that enables us to lose less money on our – using our match program." *Massachusetts Mobile Market Key Informant*
Reach/penetration of target market; weather limitations	Extend market season while adapting to climate constraints	Operate nearly year-round (40-week season) in a cold climate but shift to a lighter market schedule for half of their season to adjust for the limitations that come with a colder climate	"During November through April we do four stops per week in our lighter [season]…and there's less produce variety then and we're also going through the food hub for that time…. our light season was an additional 20 weeks this year, but our full [season is] 16 stops a week [and] is 20 weeks." *Massachusetts Mobile Market Key Informant*
Limited organizational capacity for nutrition education	Navigate local regulations to facilitate regular nutrition education offered by mobile market organization	Obtain appropriate food handlers’ cards, permitting, and investing in any necessary upgrades (e.g., hand washing station) to permit conducting nutrition education and food demonstrations	"I've asked two of our staff members to [obtain] the food handler’s license…we have to have the hand washing stations on site. And so, we had to get a temporary event permit which is for those hands-on demo and then a food service with a limited menu permit." *New Mexico Mobile Market Key Informant*
Limited organizational capacity for nutrition education; community engagement issues	Adopt a train-the-trainer model for nutrition education	Train community members to lead monthly 90-min nutrition classes within host sites that are partnering with mobile market	"So, we actually have—we do a train to trainer model to—so we engage with community, and we train the community, some community residence to be the co-leaders of those nutrition education process." *Louisiana Mobile Market Key Informant*
Expensive business model; lack of financial sustainability; limited organizational capacity for nutrition education	Incentivize purchases of several produce items that can be incorporated into a recipe	Offer a reduced cost bundle purchase that includes produce items and other ingredients that can be incorporated into a recipe that is provided	"We do a bundle, so it’s all connected with our recipe demo…. we choose the recipe for the week, [and] all of our volunteers cook that recipe at every market and then we sell a recipe bundle where you can buy all of the ingredients for that recipe…. we give them the recipe obviously also. We increased by something ridiculous like 500% last year from where we were the year prior with the sales of our recipe bundles because we were just more consistent with it." *Pennsylvania Mobile Market Key Informant*
Data collection and evaluation issues	Facilitate participation in data collection efforts among customers	Incentivize customers to complete a self-administered paper survey at the market with a gift card or a discount at the mobile market	"In return for customers filling out the survey, they get a loyalty card so every week they get $2 off a purchase at a market throughout the season." *DC Mobile Market Key Informant*
Data collection and evaluation issues	Facilitate participation in data collection efforts among customers; reduce burden on customers	As an alternative to administering a paper survey, rapid market assessment where a poster board with 1–2 multiple choice questions is displayed and customers place stickers next to their chosen answer	"A couple of times a season we do surveys… a dot survey method of data gathering…it takes three seconds or [if] you do two questions, and it takes six seconds. It gives me pretty valuable feedback. We ask a whole smattering of things like, 'How did you get here? And what do you like most about the market? And what would you like to see change?' " *Virginia Mobile Market Key Informant*

## Discussion

This paper summarizes the shared operational challenges among established mobile market organizations in the U.S. Past research indicates that lower-income individuals may have limited awareness and reluctance to trust mobile markets [8, 10], and this aligns with our finding that organizations experience challenges with engaging communities and securing trust and “buy-in.” Robinson et al. similarly cited the struggle among organizations to reconcile the desire to meet the needs of communities with the need to be strategic in where they choose to establish sites to ensure that they yield sufficient sales or volume to be economically viable. Lack of infrastructure (e.g., storage, refrigeration) and the negative impact of climate and seasonality on produce procurement have also been previously reported [13].

Our findings support past concerns surrounding how well organizations are reaching low-income populations [13]. Robinson et al. described this finding within the context of mobile markets serving too low a number of customers to make a meaningful impact on food disparities or to be financially sustainable [13]; whereas the KIs in the present study described their limited reach in terms of subsets of the low-income population they may not be reaching (e.g., non-SNAP customers). Since the time of the KI interviews, our ongoing work with mobile market operators has revealed new challenges related to community reach as a result of the COVID-19 pandemic. Operators have described the need for online ordering systems that are integrated with POS software, and text-based ordering systems to enable organizations to serve those without internet or smartphones. Robinson et al. also reported an inability to expand mobile market operations due to a lack of capacity or competition with other mobile markets [13]. Similarly, we found that many of the challenges reported here were hindrances to growing their operations despite demand from communities to expand.

This research raises the question of what financial sustainability means within the context of mobile markets. For example, how does a mobile market organization reconcile their mission (e.g., improve food access through the sale of reduced cost produce) with entrepreneurship and good business practices? Our finding that market sales are unable to cover operating expenses, thus leading organizations to seek out external funding often through grant or foundation money, supports past research with mobile markets [2, 12, 13]. Past research also highlights this conflict between a mobile market’s mission and its economic viability and questions what sustainability looks like for a mobile market organization [12, 13]. Many KIs interviewed in the present study desire financial autonomy and feel the ideal financial model would be that sales sustain the mobile market; unfortunately, the needed changes (e.g., raise prices, lower wages, reduce staff, purchase non-local produce, etc.) would undermine their mission. Several organizations accept that self-sustainability is not possible with a mobile market and have no qualms with depending on supplemental funding as long as they are fulfilling their mission. However, some organizations continue to work toward becoming less reliant on supplemental funds, even if progress is incremental. One KI expressed that they have “a long way to go” to be truly financially sustainable.

Several KIs question whether mobile markets are intended to be a long-term solution or if successful sites should be transitioned to a brick-and-mortar food retailer. One KI from a New York mobile market suggested volunteers or champions from the community could spearhead the establishment of a brick-and-mortar store or farmers’ market, but they have not had success in empowering the community to do so. Research has indicated that merely improving the food retail environment in underserved communities (i.e., opening a grocery store) may not translate to improvements in healthy food purchasing and consumption [19]. However, the community-engaged process of selecting host sites coupled with a mobile market’s longstanding presence and success in a community may have a decidedly different outcome if transitioned to a permanent retailer. Furthermore, the introduction of a new mobile market generally leads to a positive effect on F&V consumption compared to no effect by introducing a larger supermarket [3].

This research's limitations include that these challenges may not be wholly representative of all mobile market organizations given that we recruited more established organizations that serve predominantly urban regions. Therefore, operational challenges unique to more nascent organizations may differ. Also, the remedial practices we presented may not be suitable or available to newer mobile markets. In addition, although CFIR is flexible and intended to be used across all phases of evaluation, including formative evaluation and capacity/needs assessments, this research was not designed to assess an organization’s capacity in relation to an innovation. Despite this, applying CFIR during data analysis provides meaningful information on potential barriers to implementation of an evidence-based intervention (EBI). Furthermore, a systematic review of the use of CFIR found that the majority of studies that used CFIR applied the framework during- or post- implementation and the authors identified a need for the use of CFIR prior to innovation implementation to help inform implementation efforts [17].

## Conclusions

### Implications for implementation of evidence-based mobile market interventions

Barriers to implementation undermine the potential public health impact and reach of mobile markets; these limitations have implications for the successful implementation of evidence-based mobile market models [4, 5]. As evidence-based mobile market models, such as the Veggie Van model [5] are disseminated and implemented nationwide [20], it is important to understand the inner and outer setting factors that may affect successful implementation. The practices presented here can potentially prevent or counter operational challenges; but great attention should also be paid toward an organization’s points of weakness when considering implementing an EBI at a mobile market. Our findings on the CFIR domains and constructs highlight the most salient factors to implementation of a mobile market intervention. For example, staffing and funding barriers (structural characteristics), limited resources (readiness for implementation), and low receptiveness to change (implementation climate) will likely remain persistent challenges upon introduction of an EBI and may undermine its effectiveness. Therefore, strategies are needed to help overcome these challenges in order to preserve the effectiveness of EBIs, including the Veggie Van mobile market model.

### Implications for future research and practice of mobile markets

Beyond the practices described here, organizations will benefit from additional strategies that help overcome barriers while maximizing the mobile market's effectiveness and longevity. Namely, assistance with financial planning and achieving a point of financial sustainability is a high priority among organizations. The majority of organizations interviewed do not have a formal business plan or the existing plan needs updating, highlighting the potential need for business advisement. Guidance on establishing more streamlined pricing systems and identifying the most viable sites can help organizations work toward financial sustainability while maintaining affordability and adequate reach to target communities.

Many challenges that mobile market organizations face center on limited capacity, leading to constraints on necessary resources (e.g., staffing, vehicle). Securing additional funding may help to increase capacity, but innovative strategies are needed to build capacity without the need for external funding. For example, establishing community ambassador programs can build staffing capacity. Options that minimize vehicle expenses, such as leasing arrangements and permitting grant funds to be spent on vehicle maintenance, ought to be widely available and utilized.

Balancing an organization’s mission with customer demand and regional availability of produce can be an intractable struggle that undermines an organization’s ability to be sustainable. External funding may allow an organization to absorb more of the loss when selling produce at a reduced cost, but several practices (e.g., differential pricing, corporate partnerships) aimed at self-subsidizing operations may reduce an organization’s reliance on outside funding; however, it remains to be seen if these strategies are effective in the long-term. Sustainability analyses of different mobile market models may help elucidate how organizations can increase the likelihood of sustaining operations. In addition, the regional policy landscape should be explored to ensure that organizations interested in crop production to supply produce to their mobile market are unencumbered to do so. Solutions that make local produce more accessible and affordable to mobile markets, such as food hubs, may also help organizations uphold their mission while ensuring affordability.

Organizations may also need support in navigating policy related to food demonstrations, parking, and nutrition assistance and incentive programs. Food policy councils may serve as a resource for navigating and updating local policies to reflect the unique nature of mobile markets which are neither farmers’ markets or food trucks [21]. Furthermore, we ought to advocate for expansion of healthy food incentive programs (e.g. SNAP matching) to novel retail options such as mobile markets and improvement of program delivery. The Healthy Incentives Program (HIP) serves as a unique case study of a well-received and streamlined SNAP matching program implemented in Massachusetts; one KI reported an unintended consequence of overutilization leading to long lines and selling out of produce at mobile markets. This highlights the immense potential of mobile markets coupled with a well-designed incentive program to enhance these programs' reach and utilization. Furthermore, mobile market research evaluating the impact of offering SNAP at a mobile market indicates that offering financial incentives leads to increased F&V purchasing and consumption [22, 23].

The findings on CFIR constructs will guide the selection and inclusion of domains and constructs in the development of an interview guide for implementation interviews with organizations implementing the Veggie Van model. They may also be relevant to implementation of other food access interventions with community-based organizations. Use of appropriate constructs will ensure that the most relevant factors are explored, and the selection of domains/constructs used in future data collection and analyses can be properly justified, which is lacking in the available research utilizing CFIR [17].

Future research should explore the factors that impact communities’ awareness and willingness to utilize a mobile market. Organizations may need support to better reach those that are less aware or less willing to visit a mobile market. Furthermore, demonstrating the benefits of advisory boards and providing organizations with guides and resources to facilitate their establishment may enhance market reach and engagement with communities. Conducting interviews with policy makers and mobile market organization’s board members could affirm and expand on challenges and potentially offer solutions. Lastly, supporting mobile market entrepreneurs (e.g., small business development loans) that identify with the communities served can enhance the representativeness of the organization, cultivate trust with communities, and shift power and wealth imbalances in the food system.

## Data Availability

The data used during the current study are available from the corresponding author on reasonable request.

## Abbreviations

F&V: Fruit and vegetable
KI: Key informant
CFIR: Consolidated Framework for Implementation Research
EBI: Evidence-based interventions
VV: Veggie Van
SNAP: Supplemental Nutrition Assistance Program
WIC: Women, Infants, and Children
FMNP: Seniors Farmers Market Nutrition Program
POS: Point-of Sale
MOU: Memorandum of understanding
HIP: The Healthy Incentives Program

## References

[1] Hsiao BS, Sibeko L, Troy LM (2019). A systematic review of mobile produce markets: facilitators and barriers to use, and associations with reported fruit and vegetable intake. J Acad Nutr Diet.

[2] Kasprzak C, Schoonover J, Gallicchio D, Haynes-Maslow L, Vermont L, Ammerman A (2021). Using common practices to establish a framework for mobile produce markets in the United States. J Agric Food Syst Community Dev.

[3] Hollis-Hansen K, Vermont L, Zafron ML, Seidman J, Leone L (2019). The introduction of new food retail opportunities in lower-income communities and the impact on fruit and vegetable intake: a systematic review. Transl Behav Med.

[4] Gans KM, Risica PM, Keita AD, Dionne L, Mello J, Stowers KC (2018). Multilevel approaches to increase fruit and vegetable intake in low-income housing communities: final results of the 'Live Well, Viva Bien' cluster-randomized trial. Int J Behav Nutr Phys Act.

[5] Leone LA, Tripicchio GL, Haynes-Maslow L, McGuirt J, Grady Smith JS, Armstrong-Brown J (2018). Cluster randomized controlled trial of a mobile market intervention to increase fruit and vegetable intake among adults in lower-income communities in North Carolina. Int J Behav Nutr Phys Act.

[6] Gary-Webb TL, Bear TM, Mendez DD, Schiff MD, Keenan E, Fabio A (2018). Evaluation of a mobile farmer's market aimed at increasing fruit and vegetable consumption in food deserts: a pilot study to determine evaluation feasibility. Health Equity.

[7] Horning ML, Alver B, Porter L, Lenarz-Coy S, Kamdar N (2021). Food insecurity, food-related characteristics and behaviors, and fruit and vegetable intake in mobile market customers. Appetite.

[8] Kasprzak CM, Sauer HA, Schoonover JJ, Lapp MM, Leone LA (2020). Barriers and facilitators to fruit and vegetable consumption among lower-income families: matching preferences with stakeholder resources. J Hunger Environ Nutr..

[9] Haynes-Maslow L, Auvergne L, Mark B, Ammerman A, Weiner BJ (2015). Low-income individuals' perceptions about fruit and vegetable access programs: a qualitative study. J Nutr Educ Behav.

[10] Zepeda L, Reznickova A, Lohr L (2014). Overcoming challenges to effectiveness of mobile markets in US food deserts. Appetite.

[11] Lucan SC (2019). Local food sources to promote community nutrition and health: storefront businesses, farmers' markets, and a case for mobile food vending. J Acad Nutr Diet.

[12] Weissman E, Robinson J, Cecio W (2020). The promise and pitfalls of mobile markets: an exploratory survey of mobile food retailers in the United States and Canada. Agric Hum Values..

[13] Robinson J, Weissman E, Adair S, Potteiger M, Villanueva J (2016). An oasis in the desert? The benefits and constraints of mobile markets operating in Syracuse, New York food deserts. Agric Hum Values.

[14] Zepeda L, Reznickova A (2016). Potential demand for local fresh produce by mobile markets.

[15] Durlak JA, DuPre EP (2008). Implementation matters: a review of research on the influence of implementation on program outcomes and the factors affecting implementation. Am J Community Psychol.

[16] Damschroder LJ, Aron DC, Keith RE, Kirsh SR, Alexander JA, Lowery JC (2009). Fostering implementation of health services research findings into practice: a consolidated framework for advancing implementation science. Implement Sci.

[17] Kirk MA, Kelley C, Yankey N, Birken SA, Abadie B, Damschroder L (2016). A systematic review of the use of the consolidated framework for implementation research. Implement Sci.

[18] Greenhalgh T, Robert G, Macfarlane F, Bate P, Kyriakidou O (2004). Diffusion of innovations in service organizations: systematic review and recommendations. Milbank Q.

[19] Cummins S, Flint E, Matthews SA (2014). New neighborhood grocery store increased awareness of food access but did not alter dietary habits or obesity. Health affairs (Project Hope).

[20] Leone LA, Kasprzak CM., Lally A, Haynes-Maslow L, Vermont LN, Horrigan-Mauer C, Berhalter-Tumiel LM, Ammerman AS, Raja S. A novel process to recruit community partners for a hybrid implementation-effectiveness study. progress in community health partnerships : research, education, and action. In Press. 10.1353/cpr.2023.0021 PMC10569409 37462585

[21] Clayton ML, Frattaroli S, Palmer A, Pollack KM (2015). The role of partnerships in U.S. food policy council policy activities. PLoS ONE.

[22] Lyerly R, Rummo P, Amin S, Evans W, Cohen ED, Lawson E (2020). Effectiveness of mobile produce markets in increasing access and affordability of fruits and vegetables among low-income seniors. Public Health Nutr.

[23] Rummo PE, Lyerly R, Rose J, Malyuta Y, Cohen ED, Nunn A (2021). The impact of financial incentives on SNAP transactions at mobile produce markets. Int J Behav Nutr Phys Act.

